# Optical Control of Actin Network Assembly on the Supported Lipid Bilayer

**DOI:** 10.21769/BioProtoc.5656

**Published:** 2026-04-20

**Authors:** Kei Yamamoto, Makito Miyazaki

**Affiliations:** 1 RIKEN Center for Integrative Medical Sciences, 1-7-22 Suehiro-cho, Tsurumi-ku, Yokohama, Kanagawa, Japan; 2 RIKEN Center for Biosystems Dynamics Research, 2-2-3 Minatojima-minamimachi, Chuo-ku, Kobe, Hyogo, Japan; 3 Graduate School of Medicine, Science and Technology, Shinshu University, 3-1-1 Asahi, Matsumoto, Nagano, Japan; 4 PRESTO, JST, 4-1-8 Honcho, Kawaguchi, Saitama, Japan

**Keywords:** Optogenetics, Actin cytoskeleton, Lipid bilayer, In vitro reconstitution, Light-induced dimerization

## Abstract

The spatiotemporal dynamics and density of actin networks are key determinants of actin cytoskeleton–mediated cellular functions. In vitro reconstitution systems have been widely used to study actin cytoskeletal dynamics; however, many existing approaches offer limited flexibility in controlling the geometry, thickness, and density of the assembled actin networks. Here, we present an in vitro optogenetic protocol that enables precise control of actin network assembly on supported lipid bilayers using an improved light-induced dimer (iLID)-SspB-based light-inducible dimerization system. In this system, His-mEGFP-iLID is anchored to a Ni-NTA-containing lipid bilayer, while SspB-mScarlet-I-VCA, a nucleation-promoting factor fused with SspB, together with other actin cytoskeletal proteins, is supplied in bulk solution. Upon blue light illumination, SspB-mScarlet-I-VCA is recruited to the membrane in a spatially and temporally defined manner, inducing localized actin polymerization. By tuning illumination patterns and duration, actin networks with defined density, thickness, and geometry can be generated, and polymerization can be rapidly halted by stopping illumination. This protocol provides a versatile platform for reconstructing actin networks with controlled spatial organization and density, enabling quantitative analysis of density-dependent interactions between actin networks and actin-binding proteins.

Key features

• Actin networks with varying densities and arbitrary shapes can be formed on the same supported lipid bilayer by controlling blue light illumination through the objective lens.

• Actin polymerization can be stopped simply by turning off blue light illumination, enabling the formation of actin networks with defined thicknesses.

• This protocol requires purified actin and actin-binding proteins.

## Graphical overview


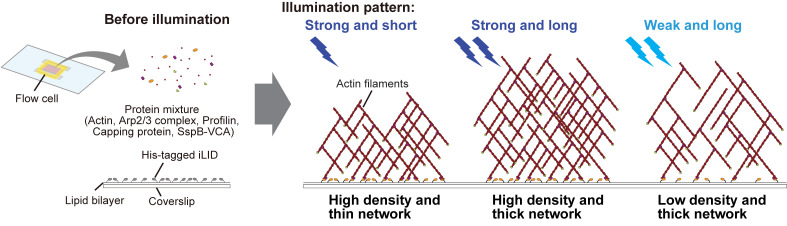


## Background

The dynamic network of the actin cytoskeleton underlies a wide range of cellular functions, including the lamellipodia, blebs, and contractile rings [1,2]. The formation of these specific structures relies on interactions between actin filaments and actin-binding proteins [3–6]. To understand the functions of actin-binding proteins, in vitro reconstitution systems using purified proteins—where actin is mixed with actin-binding proteins—have been widely employed. In particular, to mimic actin polymerization under physiologically relevant conditions, nucleation-promoting factors (NPFs) need to be spatially confined to two-dimensional substrates such as glass surfaces or lipid membranes [7,8]. Patterned UV illumination on polyethylene glycol (PEG)-coated glass has enabled the immobilization of NPFs on glass surfaces with arbitrary geometries [9–13]. Furthermore, techniques for forming spatially patterned lipid membranes have also been reported [14,15]. These techniques have enabled spatial patterning of NPFs under conditions that are closer to the in vivo environment by eliminating the height gap between PEG molecules and lipid membranes, reducing binding of NPFs with random orientation to the substrate, reducing energetic instability of lipid membranes at PEG–lipid boundaries, and minimizing heterogeneous lateral diffusion of lipid molecules [15]. However, it remains challenging to stop actin polymerization during observation, to design more complex three-dimensional structures, or to generate actin networks with different densities on the same lipid membrane. Thus, further flexibility in controlling actin polymerization is still required in in vitro reconstitution systems.

In recent years, optogenetic approaches have been increasingly applied in in vivo experimental systems using living cells and tissues to manipulate actin cytoskeleton dynamics with high spatial and temporal resolution. Among these approaches, a light-induced dimerization system, iLID-SspB, has been widely used due to its rapid association upon blue light illumination and reversible dissociation in dark conditions [16–21]. Taking this advantage, many iLID-SspB-based systems are designed so that iLID is localized to the plasma membrane or organelle membranes, and blue light illumination activates iLID to recruit SspB-fused actin cytoskeletal regulators from the cytoplasm onto the lipid membrane. The increased density of these regulatory factors on the membrane subsequently alters downstream actin cytoskeletal dynamics. Another advantage of the iLID-SspB system is that the proteins are relatively easy to purify compared to other optogenetic proteins, making them well-suited for incorporation into in vitro reconstitution assays [22,23].

Here, we present a protocol that combines actin cytoskeleton regulatory proteins with the iLID-SspB system to induce light-dependent assembly of actin networks on a lipid bilayer that mimics the plasma membrane. To generate Arp2/3 complex-mediated branched actin networks, we utilize the VCA domain located at the C-terminus of one of the major NPF, WAVE [24–26]. The V and CA domains are known to interact with G-actin and the Arp2/3 complex, respectively, inducing actin branching [24]. In this protocol, His-tagged iLID is pre-anchored to lipid membranes containing Ni-NTA, and the SspB-fused VCA domain is recruited from bulk solution onto the membrane in a light-dependent manner, thereby increasing VCA density on the membrane. Because the accumulated VCA domains are positioned in close proximity to each other, the stochastic binding of two VCA domains with Arp2/3 complexes and fragments of actin filament in bulk solution is facilitated, triggering actin polymerization [27,28]. According to previous studies on actin network reconstitution, profilin and capping protein are mixed together with the Arp2/3 complex and SspB-fused VCA [9,11,29]. Profilin and capping protein suppress actin nucleation in bulk solution and prevent excessive filament elongation, respectively. Collectively, under these conditions, light illumination triggers the assembly of actin networks in a light-dependent manner [30]. Using this system, both the shape and density of actin networks can be precisely controlled by modulating the pattern and intensity of light. Furthermore, actin polymerization can be immediately stopped by turning off the illumination. Thus, this protocol provides enhanced flexibility for actin network reconstitution and can be applied to studies of actin-binding protein function in a density-dependent manner [30], as well as to the design of three-dimensional structures of biomolecules.

## Materials and reagents

**Purified proteins**

1. His-mEGFP-iLID (see [30] for the purification method)

2. SspB-mScarlet-I-VCA (see [30] for the purification method)

3. Actin (10% labeled with Alexa Fluor 647) (see [30,31] for the purification method)

4. Arp2/3 complex (Cytoskeleton, catalog number: RP01P)

5. Profilin (see [30,32] for the purification method)

6. Capping protein (CP) (see [30] for the purification method)

*Note: Plasmids required for protein expression are available from the authors upon request. Plasmids used for the expression of His-mEGFP-iLID and SspB-mScarlet-I-VCA will be deposited at Addgene.*

**Reagents**

1. 18:1 DGS-NTA(Ni) [DGS-NTA(Ni)] (Avanti, catalog number: 790404); product format: 10 mg/mL in chloroform

2. 16:0–18:1 PC (POPC) (Avanti, catalog number: 850457); product format: powder

3. Chloroform (Wako, catalog number: 038-02606)

4. KCl (Wako, catalog number: 163-03545)

5. NaCl (Wako, catalog number: 191-01665)

6. Na_2_HPO_4_ (Wako, catalog number: 194-02875)

7. KH_2_PO_4_ (Wako, catalog number: 166-04255)

8. 99.5% ethanol (Wako, catalog number: 057-00451)

9. NaOH (Wako, catalog number: 194-18865)

10. HEPES (Sigma, catalog number: H3375)

11. EGTA (Wako, catalog number: 342-01314)

12. MgCl_2_ (Wako, catalog number: 136-03995)

13. Riboflavin 5′-monophosphate sodium salt hydrate (FMN) (Sigma, catalog number: F8399)

14. Creatine phosphokinase from rabbit muscle (CPK) (Sigma, catalog number: C3755)

15. Phosphocreatine disodium salt hydrate (Pcr) (Sigma, catalog number: P7936)

16. Adenosine 5’-triphosphate disodium salt n-hydrate (ATP) (Wako, catalog number: 019-09672)

17. (+/-)-Dithiothreitol (DTT) (Wako, catalog number: 042-29222)

18. (±)-6-Hydroxy-2,5,7,8-tetramethylchromane-2-carboxylic acid (Trolox) (Sigma, catalog number: 238813)

19. KOH (Wako, catalog number:168-21815)

**Solutions**

1. 1× PBS, pH 6.5 (see Recipes)

2. Glass washing solution (see Recipes)

3. 10× A50 buffer (see Recipes)

4. SspB mixture (see Recipes)

5. 20× energy mix (see Recipes)

6. Polymerization mixture (see Recipes)

**Recipes**

**1. 1× PBS, pH 6.5**

**Table:** 

Reagent	Final concentration	Quantity or volume
NaCl	137 mM	8 g
KCl	2.7 mM	0.2 g
Na_2_HPO_4_	8.1 mM	1.15 g
KH_2_PO_4_	1.5 mM	0.2 g
Total		1 L

Mix all reagents and adjust the pH to 6.5 using HCl. Adjust the final volume to 1 L with Milli-Q water.

**2. Glass washing solution**

**Table:** 

Reagent	Final concentration	Quantity or volume
100% EtOH	80%	800 mL
NaOH	50 g/L	50 g
Total		1 L

Mix the reagents and adjust the final volume to 1 L with Milli-Q water.

**3. 10× A50 buffer**

**Table:** 

Reagent	Final concentration	Volume (for 1 L)
1 M HEPES-KOH, pH 7.6	0.5 M	500 mL
2 M KCl	0.5 M	250 mL
1 M MgCl_2_	50 mM	50 mL
0.2 M EGTA	10 mM	50 mL
Total		1 L

Mix the reagents and adjust the final volume to 1 L with Milli-Q water.

**4. SspB mixture**

**Table:** 

Reagent	Final concentration	Volume (for 20 μL)
20 mM Trolox in A50 buffer	2 mM	2 μL
200 μM FMN in A50 buffer	10 μM	1 μL
4.3 μM SspB-mScarlet-I-VCA in A50 buffer	150 nM	0.7 μL
1× A50 buffer		16.3 μL
Total		20 μL

Before mixing all components required for actin polymerization, we recommend first confirming that SspB-mScarlet-I-VCA is recruited to the lipid membrane upon light illumination in a simple composition without actin cytoskeletal proteins.

**5. 20× energy mix**

**Table:** 

Reagent	Final concentration	Volume (for 10 μL)
0.2 M ATP in A50 buffer	20 mM ATP	1 μL
0.4 M Pcr in A50 buffer	0.2 M Pcr	5 μL
10 mg/mL CPK in A50 buffer	2 mg/mL CPK	2 μL
1 M DTT in A50 buffer	0.2 M DTT	2 μL
Total		10 μL

The 20× energy mix is not stable for long-term storage and should therefore be freshly prepared for each experiment.

**6. Polymerization mixture**

**Table:** 

Reagent	Final concentration	Volume (for 20 μL)
2× A50 buffer		2 μL
20 mM Trolox in A50 buffer	2 mM	2 μL
20× energy mix	1×	1 μL
200 μM FMN in A50 buffer	10 μM	1 μL
200 μM profilin in A50 buffer	15 μM	1.5 μL
250 nM CP in A50 buffer	25 nM	2 μL
2.8 μM Arp2/3 complex in A50 buffer	100 nM	0.7 μL
4.3 μM SspB-mScarlet-I-VCA in A50 buffer	150 nM	0.7 μL
50 μM actin in G-buffer	5 μM	2 μL
1× A50 buffer		7.1 μL
Total		20 μL

To minimize actin polymerization in bulk solution, keep all solutions on ice and add actin last. Mix all reagents within 5 min and immediately load into a flow cell. Dilute capping protein (CP) immediately before use from a purified stock solution (30–50 μM) to a concentration of 250 nM in A50. Add the other proteins directly from their stock solutions, with mixing volumes adjusted according to stock concentrations. Adjust the total reaction volume to 20 μL with A50 buffer. Add 2× A50 buffer in a volume equal to that of actin.

**Laboratory supplies**

1. 1.5 mL glass tube (Maruemu, catalog number: 0407-03)

2. 24 × 60 mm coverslip (Matsunami, catalog number: C024601)

3. 18 × 18 mm coverslip (Matsunami, catalog number: C018181)

4. Double-sided tape; thickness: 0.3 mm (Scotch, catalog number: PBW-20)

5. VaLaP (Wako, catalog numbers: 224-00165, 128-00115, and 167-13335)

6. Membrane filter (Cytiva, catalog number: 10419504)

7. Filter support (Cytiva, catalog number: 230300)

8. Pipette tips (10 μL) (Eppendorf, catalog number: 0030000811)

9. Pipette tips (200 μL) (Eppendorf, catalog number: 0030000870)

10. Pipette tips (1,000 μL) (Eppendorf, catalog number: 0030000935)

11. 1.5 mL tube (Eppendorf, catalog number: 022363247)

12. Parafilm

## Equipment

1. Vacuum desiccator (AS ONE, catalog number: 1-5801-23-20)

2. Glove bag (AS ONE, catalog number: 3-118-11)

3. Nitrogen gas line

4. Heat sealer (Fuji Impulse, model: P-300)

5. Vacuum sealing machine and associated bags and rolls (MagicVac, catalog number: V952S)

6. Vortex

7. Liquid nitrogen tank (Jecc Torisha, model: Cebell5)

8. Water bath (TAITEC, model: EX-B)

9. Microscope (Nikon, model: ECLIPSE Ti2)

10. Digital micromirror device (DMD) module (Nikon, model: TI-LA-DMD)

11. Objective lens (Nikon, model: CFI Plan Lamda D 60×, N.A. 1.42)

12. EMCCD camera (Andor, model: iXon Life 888)

13. Light source for DMD (Lumencor, model: SOLA)

14. Spinning disk confocal unit (Yokogawa Electric Corporation, model: CSU-W1)

15. ND filter (Thorlabs, catalog number: NE220B)

16. Dark microscopy room with red room light

17. Power sensor (Advantest, model: TQ8210)

18. Glass slide staining dish with stainless storage rack

19. Sonicator (AS ONE, catalog number: AS12GTU)

20. Plasma cleaner (Harrick Plasma, catalog number: PDC-32G)

21. Hot plate (Thermo Fisher Scientific, catalog number: HP2305BQ)

22. Small brush

23. Mini-extruder (Avanti, catalog number: 610000)

24. Humidity chamber (a plastic Petri dish filled with wet filter paper)

25. Deep freezer (-80 °C)

26. Freezer (-20 to 30 °C)

27. Fridge (4 °C)

28. Eppendorf pipette kit (Eppendorf, catalog number: 2231300002)

29. Weighing scale

## Software and datasets

1. NIS-elements (Nikon, Version 5.42.03)

## Procedure

**A. Preparation of the vesicle stock solution**

1. Aliquot 132.15 μL of a 10 mg/mL (9.46 mM) DGS-NTA(Ni) solution in chloroform into glass tubes.

2. Dry the tubes overnight in a vacuum desiccator.

3. Fill the glove bag with nitrogen gas.

4. Cap the glass tubes containing the dried DGS-NTA(Ni) under a nitrogen atmosphere.

5. Aliquot 5–10 mg of POPC powder into Eppendorf tubes under a nitrogen atmosphere.

*Note: The exact mass of the powder can be determined from the difference in tube weight before and after aliquoting.*

**Figure 1.**
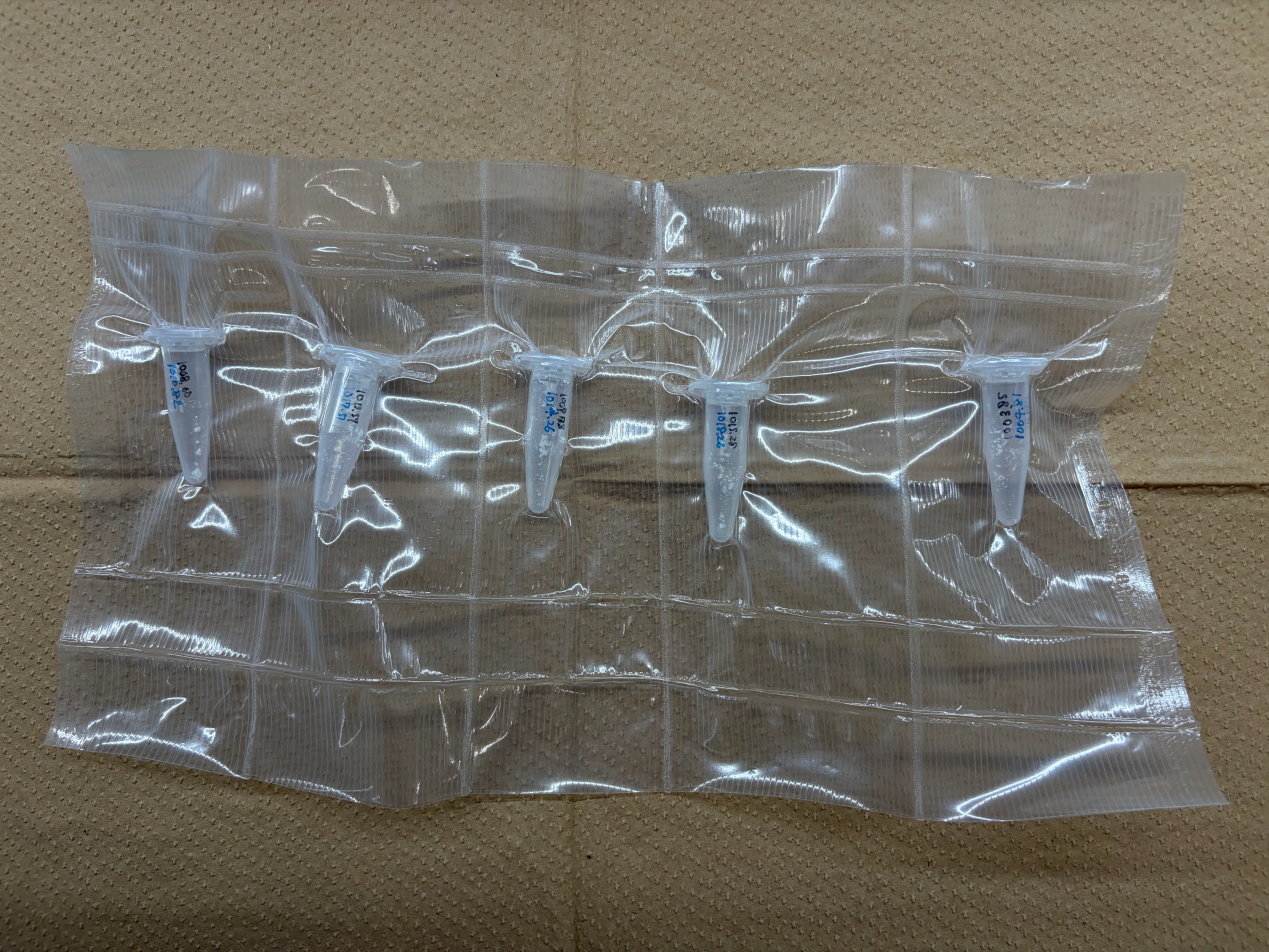
Lipid storage method. Partition a polyethylene roll into individual compartments using a heat sealer. Place one Eppendorf or glass tube into each compartment and vacuum-seal the roll.

6. Vacuum-seal the glass tubes containing a dry film of DGS-NTA(Ni) and the Eppendorf tubes containing POPC, and store in the freezer until dissolving in chloroform (Figure 1).

**Pause point:** The stored DGS-NTA(Ni) and POPC remain usable for subsequent experiments for approximately six months.

7. Add 62.5 μL of chloroform to the glass tube containing a dry film of DGS-NTA(Ni) to prepare a 20 mM solution.

8. Add chloroform to the Eppendorf tube containing POPC powder to prepare a 20 mM solution. Adjust the volume of chloroform according to the amount of powder in the tube.

*Note: Chloroform has low viscosity and evaporates readily; therefore, it should be added quickly, and the tube should be capped immediately. Pipetting chloroform in and out of the tip several times helps prevent leakage from the pipette tip.*

9. Prepare a new glass tube and mix 7.2 μL of DGS-NTA with 112.8 μL of POPC to obtain a lipid mixture containing 6% (mol/mol) DGS-NTA(Ni).

*Note: By reducing the amount of DGS-NTA(Ni) in the POPC, the ratio of DGS-NTA(Ni) to POPC can be adjusted in this step. The density of the formed actin network can be regulated not only by the intensity of light illumination but also by the density of DGS-NTA (Ni) in the lipid membrane.*

10. Prepare two new glass tubes and aliquot 50 μL of the mixture into each tube.

11. Dry the tubes overnight in a vacuum desiccator.

12. Add 1 mL of PBS (pH 6.5) to each tube and vortex for 30 s.

13. Close the tube caps and store at 4 °C overnight.

14. Transfer the vesicle solution into an Eppendorf tube.

15. Snap-freeze the Eppendorf tube in liquid nitrogen.

16. Thaw the tube in a 30 °C water bath and repeat this freeze-thaw cycle five times to obtain unilamellar vesicles.

17. Aliquot the solution and store it in the freezer as a 1 mM stock solution.

**Pause point:** The stored vesicle solution remains usable for subsequent experiments for approximately three months.

**B. Optimization of light illumination intensity**

*Note: The goal of this section is to determine the appropriate range of blue light intensity by correlating the software settings with the actual illumination intensity, thereby enabling control of actin network density through the microscope software. This section is not required if control of actin network density is not needed.*

1. Turn on the computer and microscope and launch the NIS-Elements software. The excitation laser and fluorescence filter settings are as follows: Excitation laser, 405, 488, 561, and 640 nm; dichroic mirror, DM 405/488/568/647 nm; emission filters, 447/60, 525/50, 617/73, and 685/40 nm. The filter settings for the digital micromirror device (DMD) are as follows: Dichroic mirror, DM 500 nm; excitation filter 470/40 nm; emission filter, 535/50 nm. DMD is an instrument that allows patterned light illumination by individually controlling the angles of numerous mirrors arranged on a chip. By introducing the DMD module into a light path separate from the imaging excitation light, selected regions within the field of view can be selectively illuminated.

2. Insert an ND filter into the output port of the DMD light source.

*Note: iLID is highly sensitive to blue light and, in many cases, is fully activated even at 1%–5% output in the software. Therefore, to control actin network density by modulating illumination intensity, we strongly recommend inserting an ND filter into the DMD light path. The ND filter can be placed anywhere along the light path, as long as it does not interfere with image acquisition.*

3. Make the room lights as dark as possible and fix the power sensor on top of the objective lens.

4. Calibrate the DMD light path according to the manufacturer’s protocol.

5. Assign a region of interest (ROI) through the DMD in NIS-Elements, illuminate the designated region with blue light, and measure the light intensity.

*Note: We have confirmed that the level of iLID activation can be tuned stepwise within the range of 0.04–0.18 nW/µm^2^. To determine optimal illumination conditions, we recommend generating a calibration curve by outputting various excitation intensities in the software and measuring the corresponding light intensities.*

**C. Preparation of the handmade flow cell**

**Figure 2.**
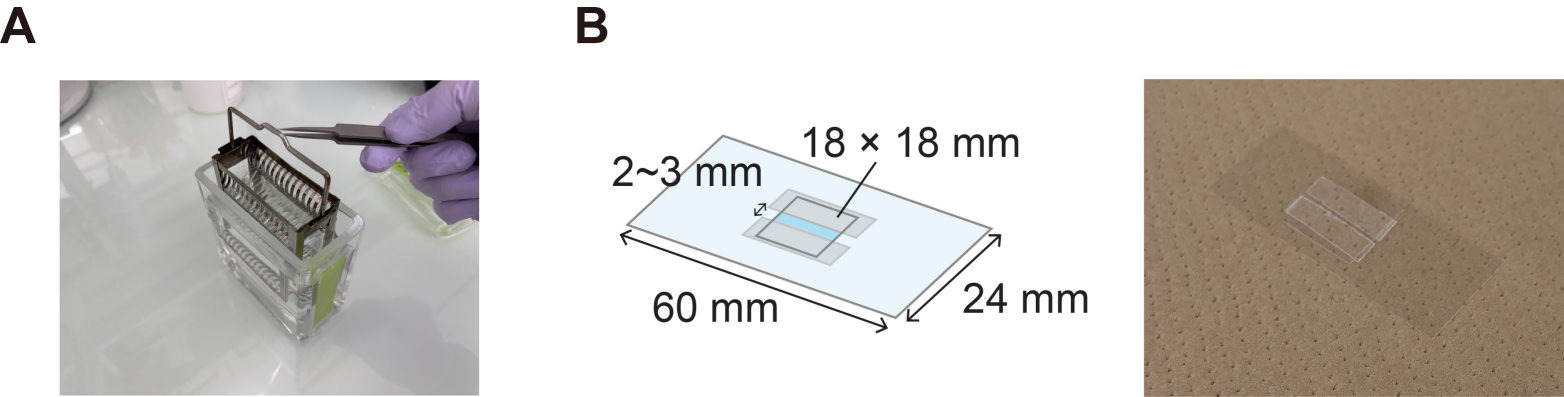
Preparation of the flow cell. (A) Glass slide staining dish with stainless storage rack. (B) Flow cell design (left) and representative image (right). The thickness of the double-sided tape is 0.3 mm.

1. Place 24 × 60 mm coverslips into a stainless storage rack and immerse the rack in a glass slide staining dish filled with glass washing solution (see Recipes; Figure 2A).

2. Place the glass slide staining dish in a sonicator and wash for 90 min.

3. Rinse the coverslips and storage rack five times with Milli-Q water.

4. Fill the glass slide staining dish with Milli-Q water and wash for 60 min.

5. Rinse the coverslips and storage rack five times with Milli-Q water.

**Critical:** After washing, confirm that the surface of the cover glasses is hydrophilic and does not repel water.

**Pause point:** The coverslips can be stored for approximately two weeks in a storage rack filled with Milli-Q water.

6. Dry the coverslips under a stream of nitrogen gas.

7. Place the coverslips in a plasma cleaner and clean for approximately 5 min.

8. Put two strips of double-sided tape with a 2–3 mm gap on the plasma-treated coverslip and place an 18 × 18 mm coverslip on top (Figure 2B).

*Note: The volume of the flow cell is approximately 10–15 μL.*

9. Melt VaLaP on a hot plate and use a brush to completely seal all areas of the channel except the inlet and outlet.

**Pause point:** The flow cell can be stored exposed to air for 2–3 days.

**D. Preparation of the iLID-bound lipid bilayer**

**Critical:** To prevent activation of iLID, perform all subsequent steps in a dark room illuminated with a red light.

1. Dilute the 1 mM vesicle solution threefold with PBS (pH 6.5) to obtain a 0.33 mM solution.

*Note: To achieve a uniformly spread lipid bilayer, optimize the volume of vesicle solution according to the area and height of the flow cell used.*

2. Assemble the mini-extruder, membrane filter, and filter support.

3. Filtrate the vesicle solution through a syringe 10 times to homogenize the vesicle size.

4. Load 20 μL of the vesicle solution into the flow cell and incubate at room temperature for 10 min.

*Note: To prevent drying of the flow cell, prepare a humidity chamber by filling a Petri dish with wet filter paper and placing a sheet of Parafilm and the flow cell on top.*

5. Wash out excess vesicle solution with 50 μL of 1× A50 buffer.

*Note: To wash the flow cell, place a pipette tip at the inlet and dispense buffer while holding a piece of filter paper at the outlet to absorb the solution inside the flow cell. During washing, avoid air bubbles in the flow cell to prevent detachment of the lipid membrane.*

**Figure 3.**
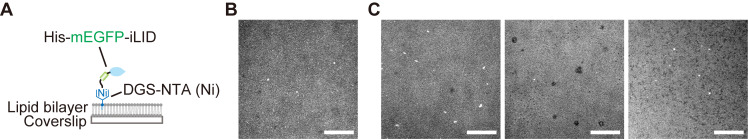
Quality check of the supported lipid bilayer. (A) Schematic illustration of improved light-induced dimer (iLID) anchored to the lipid bilayer via His-tag. (B, C) iLID signals visualized by mEGFP: (B) a smooth and uniform lipid surface; (C) aggregates of large vesicles (left) and abnormal dark spots (middle and right) in the lipid bilayer, caused by old vesicle stock samples or contaminants on coverslips. Scale bars, 20 μm.

6. Load 20 μL of His-mEGFP-iLID diluted to 7 μM into the flow cell and incubate at room temperature for 5 min.

7. Secure the flow cell on the microscope stage using VaLaP and double-sided tape.

8. Wash out excess His-mEGFP-iLID with 50 μL of 1× A50 buffer.

9. Observe the His-mEGFP-iLID signal under the microscope and confirm uniform binding on the lipid membrane surface (Figure 3).

*Note: We confirmed that the edges of the flow cell, near the double-sided tape, tend to exhibit heterogeneous lipid coating, as shown in Figure 3C. Therefore, we recommend performing imaging near the central region of the flow cell.*

**E. Light-induced actin polymerization on the lipid bilayer**

1. Quickly prepare the polymerization mixture (see Recipes) on ice.

*Note: Instead of the polymerization mixture, loading the SspB mixture (see Recipes) into the flow cell allows us to simply confirm the SspB recruitment to the lipid membrane without actin polymerization.*

2. Load the polymerization mixture into the flow cell on the microscope stage and completely seal the inlet and outlet with VaLaP.

3. Find the focal plane based on the SspB-mScarlet-I-VCA signal and turn on the perfect focus function.

4. Start imaging.

**Figure 4.**
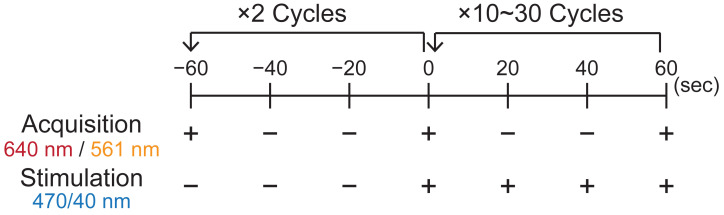
Time course of image acquisition and stimulation

*Note: To flexibly control illumination intensities, illumination on/off timing, and image acquisition intervals, the JOBS function in NIS-Elements is useful. To continuously keep SspB on the lipid membrane, apply light illumination for 500 ms at 20 s intervals (Figure 4). Recruitment of SspB to the lipid membrane occurs approximately 20 s after the onset of light illumination, followed by the initiation of actin polymerization.*

## Data analysis

Methods for quantifying the fluorescence intensity of the assembled actin networks and recruited SspB-mScarlet-I-VCA, as well as the morphology of actin networks, are described in detail in the methods section of [30]. In addition, actin network density can be estimated more quantitatively by generating a calibration curve of fluorescence intensity using filamentous actin solutions of known concentrations measured under the same microscope settings and comparing these values with the fluorescence intensity of light-induced actin networks. Because protein activity and concentration may vary slightly between experiments, we recommend performing at least three independent reconstitution experiments.

## Validation of protocol

This protocol has been used and validated in the following research article:

- Kei Yamamoto and Makito Miyazaki [30]. Optogenetic actin network assembly on lipid bilayer uncovers the network density-dependent functions of actin-binding proteins. *Nature Communications* (Figures 2–6, and Supplementary figures 2–8).
  Briefly, to validate that actin network density can be controlled by light intensity, five different illumination intensities were applied to distinct regions on the same lipid bilayer (Figure 5A). Quantitative analysis confirmed that both the fluorescence intensity of recruited SspB-mScarlet-I-VCA and subsequently polymerized actin increased in a light intensity-dependent manner (Figure 5B, C). Furthermore, by measuring the thickness of polymerized actin networks formed under different illumination durations, we demonstrated that actin network thickness can be controlled by the duration of light exposure (Figure 5D–F).
  **Figure 5.**
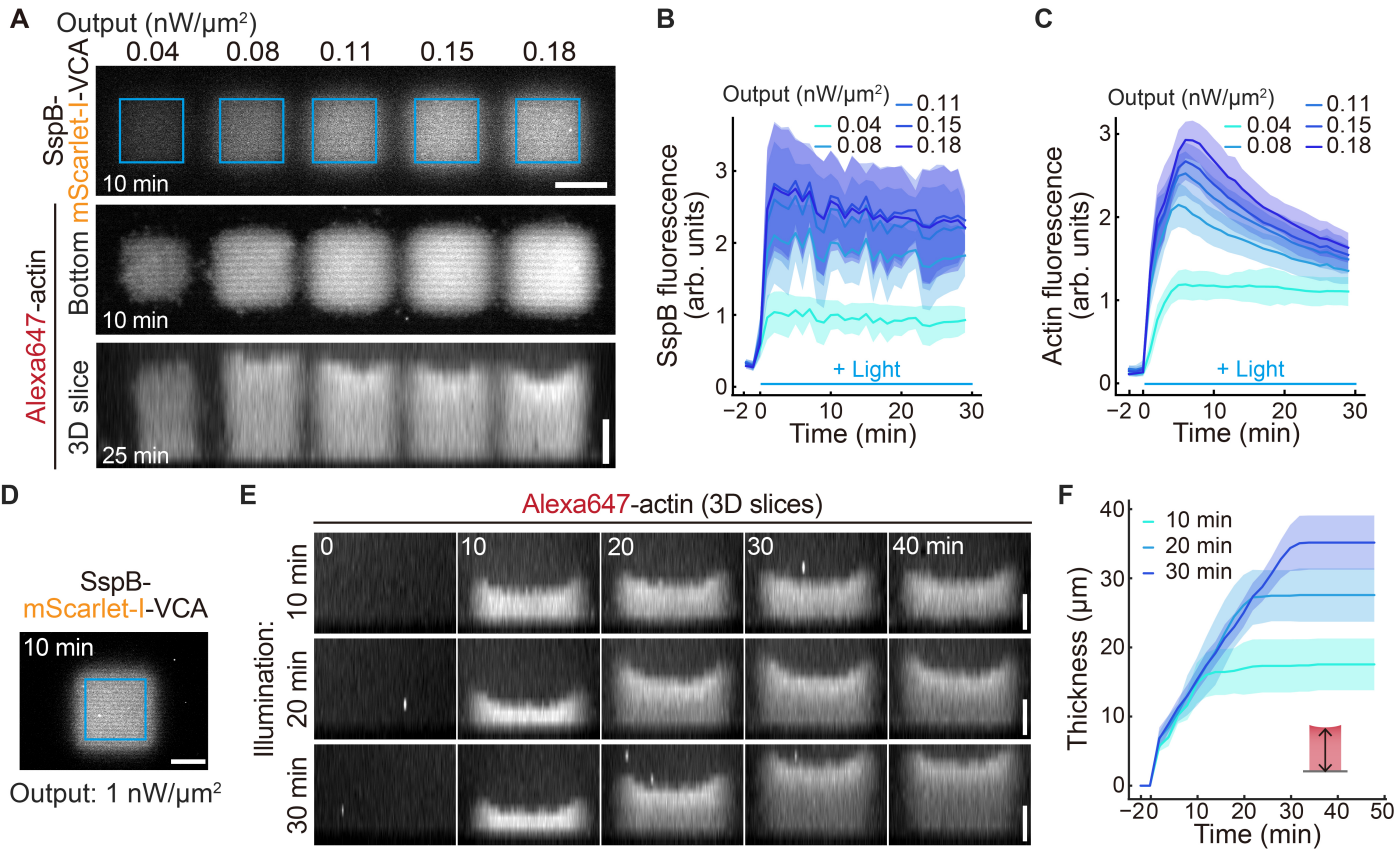
Validation of actin network assembly on the supported lipid bilayer. (A) Representative images of the recruitment of SspB-mScarlet-I-VCA (top) and subsequently polymerized actin (middle, bottom) under five different light-powered illuminations on 6% DGS-NTA(Ni) lipid at each indicated time point. Blue squares indicate the 25 × 25 μm illuminated regions. (B, C) Fluorescence intensities of SspB-mScarlet-I-VCA (B) and Alexa647-actin (C) under the five different light-powered illuminations. The mean values (bold lines) are plotted as a function of time with the SD. n = 6 for each condition. (D) Representative images of the recruitment of SspB-mScarlet-I-VCA to validate the thickness of the actin network by illumination duration. Blue square indicates the 37 × 37 μm of illuminated regions. (E) Control of actin network thickness by illumination durations. (F) Thickness of the actin network in three different illumination duration patterns. The mean values (bold lines) are plotted as a function of time with the SD. n = 12 for each condition. Scale bars, 20 μm. The figure is modified from Yamamoto K. and Miyazaki M. [30] with permission under the Creative Commons Attribution-NonCommercial-NoDerivatives 4.0 International License (http://creativecommons.org/licenses/by-nc-nd/4.0/).

## General notes and troubleshooting

**General notes**

1. In this protocol, actin networks of various densities can be formed on the same lipid bilayer by controlling light intensity. At the single-filament level, actin polymerization is triggered by the stochastic binding of two VCA domains on the lipid bilayer with Arp2/3 complexes and actin filament fragments diffusing in solution. Therefore, the resulting actin network density depends on the density of VCA domains on the lipid bilayer. Blue light illumination in the range of approximately 0.04–0.18 nW/μm^2^ allowed control of SspB-mScarlet-I-VCA accumulation in a light intensity–dependent manner. Using this experimental setup, the addition of other actin-binding proteins enables systematic investigation of their network density-dependent functions [30]. Furthermore, this approach may also be useful for studying the spatial interactions between intracellular organelles and actin networks.

2. When VCA domains are accumulated using light intensities ≥0.08 nW/μm^2^, a transient formation of a high-density actin network followed by a decrease in network density is observed. Along the z-axis of the actin pillars, this results in a density gradient, with a higher-density apical region and a lower-density basal region. This trend arises from a depletion effect, in which highly concentrated VCA domains rapidly consume actin and actin-binding proteins. Because the depletion effect is most pronounced at the center of the illuminated region, the apical surface of the actin pillar often exhibits a cup-shaped morphology with a central depression. To suppress the depletion effect and achieve a more uniform actin network density, reducing the illumination area, lowering the light intensity, or decreasing the density of DGS-NTA(Ni) in the lipid membrane is effective.

3. It is recommended to minimize phototoxicity on actin networks during blue light illumination and to avoid photobleaching from the excitation laser. In this protocol, the blue light used to induce actin polymerization is attenuated to approximately 0.04–0.18 nW/μm^2^ by inserting an ND filter into the light path. In addition, Trolox is mixed in the actin polymerization mixture, which effectively suppresses phototoxicity to actin filaments caused by reactive oxygen species. On the other hand, the laser used for imaging is relatively strong, and we observed gradual photobleaching of SspB-mScarlet-I-VCA recruited on the lipid membrane (Figure 5B). For actin, fluctuations in fluorescence intensity are predominantly due to depletion effects (Figure 5C). To minimize the effect of phototoxicity and photobleaching, we recommend performing imaging using a spinning-disk confocal unit with the lowest laser intensity and shortest exposure time.

4. In this protocol, due to the fluidity of the lipid membrane, the localization of VCA domains and the resulting actin networks leak beyond the light-illuminated regions defined in the software. Furthermore, diffusion of VCA domains from the edges of the illuminated region leads to a relatively lower actin density at the periphery of the formed network.

**Troubleshooting**

**Problem 1:** The lipid membrane does not form properly, and the His-mEGFP-iLID signal appears non-uniform.

Possible causes: The glass surface is not clean, or the vesicle solution is old.

Solutions: Use freshly cleaned glass coverslips and store them in Milli-Q water. Use plasma-treated glass within one week. Use vesicle solutions within one week after thawing and filtration.

**Problem 2:** SspB-mScarlet-I-VCA is not recruited to the lipid membrane, or its accumulation is weak.

Possible causes: The lipid membrane is not properly formed, or blue light is not being applied.

Solutions: Confirm that the lipid membrane is uniform by imaging His-mEGFP-iLID, following Problem 1. Check the microscope settings and ensure that blue light illumination is applied. In a dark room, even though blue light at intensities of 0.04–0.18 nW/μm^2^ is weak, it can be visually detected.

**Problem 3:** SspB-mScarlet-I-VCA is recruited to the lipid membrane, but actin does not polymerize.

Possible causes: The Arp2/3 complex activity is reduced, or full-length SspB-mScarlet-I-VCA is not properly purified.

Solutions: Confirm the activity of the Arp2/3 complex using a pyrene assay and use the Arp2/3 complex within a few hours after thawing. Verify the purity of SspB-mScarlet-I-VCA by SDS-PAGE. If shorter fragments are contaminated, optimize the expression conditions in *E. coli* or remove the contaminants by gel filtration.

**Problem 4:** Actin polymerization occurs upon light illumination, but the density cannot be controlled by light intensity.

Possible cause: The light intensity is too high to control the accumulation level of SspB-mScarlet-I-VCA on the lipid membrane.

Solution: First, use a power sensor to directly measure the intensity of blue light at the objective lens and estimate an appropriate intensity range. Under our experimental conditions, SspB-mScarlet-I-VCA accumulation saturates at an intensity of ~0.18 nW/μm^2^. In many cases, excessive light intensity is the primary issue; therefore, insert an ND filter in the light path so that the actual output becomes approximately 1 nW/μm^2^ when the software output is set to 100%. Next, load the SspB mixture onto the iLID-bound lipid membrane and quantify the accumulation of SspB-mScarlet-I-VCA under different illumination intensities. By generating a calibration curve, an intensity range that allows controlled accumulation of SspB-mScarlet-I-VCA can be identified. Because the density of the actin network depends on the density of VCA domains on the lipid membrane, the actin network density can be controlled within this range.

**Problem 5:** Excessive actin polymerization occurs, and nonspecific polymerization appears outside the illuminated region.

Possible causes: A fraction of the monomeric actin solution has oligomerized, CP activity is reduced, or the room is not completely dark.

Solution: Keep actin on ice for more than 10 days after thawing to ensure it is fully monomeric. Since the final concentration of CP is low (~25 nM), dilute it immediately before use to prevent adsorption to the Eppendorf tube. Completely shield the microscope stage from ambient light.
